# High-quality permanent draft genome sequence of the *Bradyrhizobium elkanii* type strain USDA 76^T^, isolated from *Glycine max* (L.) Merr

**DOI:** 10.1186/s40793-017-0238-2

**Published:** 2017-03-04

**Authors:** Wayne Reeve, Peter van Berkum, Julie Ardley, Rui Tian, Margaret Gollagher, Dora Marinova, Patrick Elia, T. B. K. Reddy, Manoj Pillay, Neha Varghese, Rekha Seshadri, Natalia Ivanova, Tanja Woyke, Mohamed N. Baeshen, Nabih A. Baeshen, Nikos Kyrpides

**Affiliations:** 1 School of Veterinary and Life Sciences, Murdoch University, Murdoch, Australia; 2 U.S. Department of Agriculture, Soybean Genomics and Improvement Laboratory, Beltsville Agricultural Research Center, 10300 Baltimore Avenue, Bldg. 006, Beltsville, MD 20705 USA; 3 Curtin University Sustainability Policy Institute, Curtin University, Bentley, WA Australia; 4 DOE Joint Genome Institute, Walnut Creek, CA USA; 5 Biological Data Management and Technology Center, Lawrence Berkeley National Laboratory, Berkeley, CA USA; 6 Department of Biology, Faculty of Science, University of Jeddah, Jeddah, Saudi Arabia; 7 Department of Biological Sciences, Faculty of Science, King Abdulaziz University, Jeddah, Saudi Arabia

**Keywords:** Root-nodule bacteria, GEBA-RNB, Nitrogen fixation, *Bradyrhizobium*, Soybean, Type III secretion system

## Abstract

*Bradyrhizobium elkanii* USDA 76^T^ (INSCD = ARAG00000000), the type strain for *Bradyrhizobium elkanii*, is an aerobic, motile, Gram-negative, non-spore-forming rod that was isolated from an effective nitrogen-fixing root nodule of *Glycine max* (L. Merr) grown in the USA. Because of its significance as a microsymbiont of this economically important legume, *B. elkanii* USDA 76^T^ was selected as part of the DOE Joint Genome Institute 2010 *Genomic Encyclopedia for Bacteria and Archaea-Root Nodule Bacteria* sequencing project. Here the symbiotic abilities of *B. elkanii* USDA 76^T^ are described, together with its genome sequence information and annotation. The 9,484,767 bp high-quality draft genome is arranged in 2 scaffolds of 25 contigs, containing 9060 protein-coding genes and 91 RNA-only encoding genes. The *B. elkanii* USDA 76^T^ genome contains a low GC content region with symbiotic *nod* and *fix* genes, indicating the presence of a symbiotic island integration. A comparison of five *B. elkanii* genomes that formed a clique revealed that 356 of the 9060 protein coding genes of USDA 76^T^ were unique, including 22 genes of an intact resident prophage. A conserved set of 7556 genes were also identified for this species, including genes encoding a general secretion pathway as well as type II, III, IV and VI secretion system proteins. The type III secretion system has previously been characterized as a host determinant for *Rj* and/or *rj* soybean cultivars. Here we show that the USDA 76^T^ genome contains genes encoding all the type III secretion system components, including a translocon complex protein NopX required for the introduction of effector proteins into host cells. While many bradyrhizobial strains are unable to nodulate the soybean cultivar Clark (*rj1*), USDA 76^T^ was able to elicit nodules on Clark (*rj1*), although in reduced numbers, when plants were grown in Leonard jars containing sand or vermiculite. In these conditions, we postulate that the presence of NopX allows USDA 76^T^ to introduce various effector molecules into this host to enable nodulation.

**Electronic supplementary material:**

The online version of this article (doi:10.1186/s40793-017-0238-2) contains supplementary material, which is available to authorized users.

## Introduction

Soybean (*Glycine max*) (L.) Merr. is the dominant and the most important commercial legume crop species, yielding food oil and animal meal as well as nutritious vegetable protein [1–3]. The plant was first introduced into USA agriculture during the mid-18th century and was mainly used as a forage crop until the 1920s [4]. The development of new cultivars, along with technological advances in soybean processing and increased demand for soybean products, has led to major increases in production during the 20th century [4].

As with most papilionoid legumes, soybean engages in a symbiotic relationship with dinitrogen-fixing soil bacteria known as rhizobia and is able to obtain on average 50–60% of its required nitrogen through symbiotic nitrogen fixation [5]. A greater understanding of the symbiosis between soybean and its cognate rhizobia is of direct relevance for maintaining environmentally sustainable high crop yields, which significantly contributes to the Sustainable Development Goals adopted in September 2015 as part of the UN’s development agenda ‘Transforming our world: the 2030 Agenda for Sustainable Development’ [6].

The soybean-nodulating bacteria, known as *Rhizobium japonicum* according to a 1929 classification scheme [7], were reclassified as *Bradyrhizobium japonicum* in 1982 because of several fundamental morphological and physiological differences with the genus *Rhizobium* [8]. The bacteria isolated from nodules of soybean had previously been shown to be phenotypically diverse, even though they were grouped together in the species *Bradyrhizobium japonicum*. One of the major methods that demonstrated this diversity was serology, which was used to classify individual isolates into 17 distinct serogroups [9]. This was accomplished by generating antisera to specific strains in the USDA collection in Beltsville and then using the sera to generate a serological scheme. One of the strains used to generate antisera was USDA 76^T^ and all isolates that cross-reacted with the antiserum generated with this serotype strain were combined together in the 76 serogroup. The strain USDA 76^T^ deposited in the Beltsville collection was a re-isolate from a greenhouse-grown plant inoculated with USDA 74 in Maryland. In turn, USDA 74 was a re-isolate of USDA 8 from a plant passage field test in California in 1956. The original parent culture of USDA 76^T^ is USDA 8, which was isolated from soybean grown at the Arlington Farm, Virginia in 1915.

Differences among the soybean root nodule bacteria classified as *B. japonicum* were also demonstrated using molecular methods. Hollis et al. [10] reported the presence of three DNA homology groupings by analysis of 28 strains within the soybean rhizobia. Using this approach, nine of the 17 serogroups were assigned to three DNA homology groupings: group I, the closely related group Ia and the more divergent group II. Supporting evidence for these three groupings was obtained by Kuykendall et al. [11]. By sequence analysis of the 16S rRNA genes, each of the 17 serotype strains representing the serogroups were also placed into three closely related groups [12] that matched their separation by DNA homology. Since soybean strains could be distinguished phenotypically and by several approaches in molecular biology, Kuykendall et al. [13] proposed that DNA homology group II strains be separated from *B. japonicum* as the species *Bradyrhizobium elkanii*, with USDA 76^T^ as the type strain.

Because of these distinguishing characteristics and its significance as a microsymbiont of the economically important legume soybean, *B. elkanii* USDA 76^T^ was selected as part of the DOE Joint Genome Institute 2010 *Genomic Encyclopedia for* *Bacteria* *and Archaea-Root Nodule Bacteria* sequencing project [14, 15]. Here we present a summary classification and a set of general features for *B. elkanii* strain USDA 76^T^, together with a description of its genome sequence and annotation.

## Organism information

### Classification and features

**Fig. 1:**
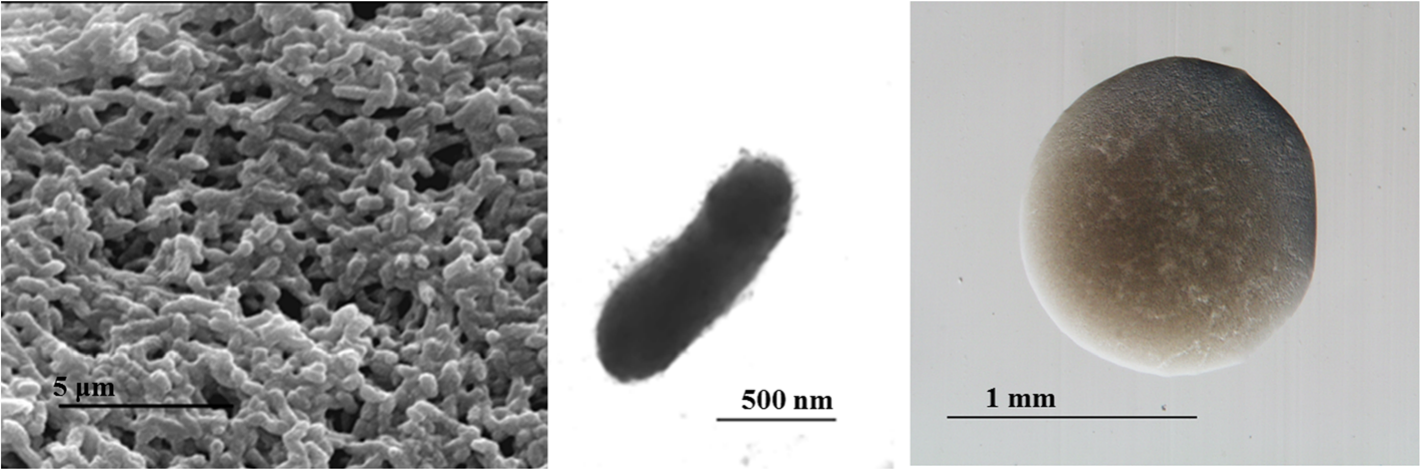
Images of *Bradyrhizobium elkanii* USDA 76^T^ using scanning (*Left*) and transmission (*Center*) electron microscopy as well as light microscopy to visualize colony morphology on solid media (*Right*)

*Bradyrhizobium elkanii* USDA 76^T^ is a motile, non-sporulating, non-encapsulated, Gram-negative strain in the order *Rhizobiales* of the class *Alphaproteobacteria*. The rod shaped form has dimensions of approximately 0.5 μm in width and 1.0–2.0 μm in length (Fig. 1 *Left* and *Center*). It is relatively slow growing, forming colonies after 6–7 days when grown on ½ Lupin Agar [16], Modified Arabinose Gluconate [17] and modified Yeast Mannitol Agar [18] at 28 °C. Colonies on ½ LA are opaque, slightly domed and moderately mucoid with smooth margins (Fig. 1 *Right*).

**Fig. 2:**
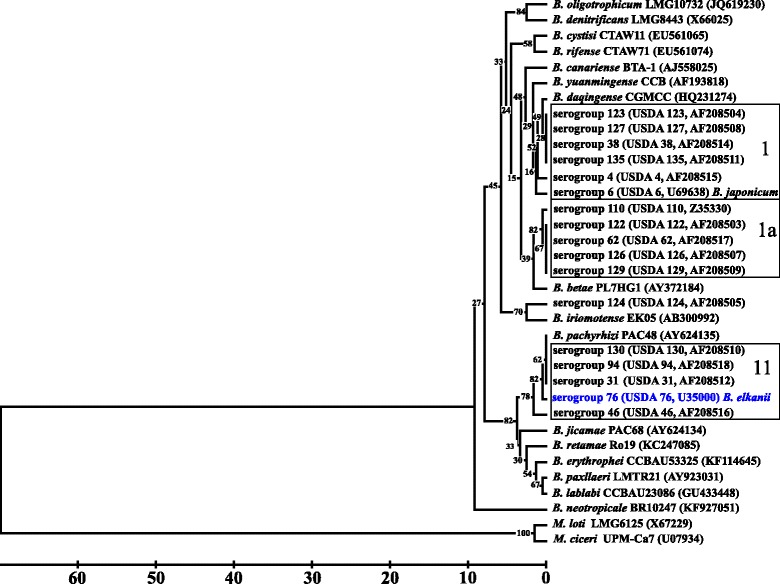
Comparison of the 16S rRNA gene of *Bradyrhizobium elkanii* USDA 76^T^ (shown in bold blue print) with those of other proposed *Bradyrhizobium* species and the serotype strains of the remaining 16 serogroups of the soybean bradyrhizobia. DNA homology affiliation of the different soybean serogroup strains are indicated within the rectangles. DNA homology values for the serogroup strains USDA 4, USDA 94, USDA 124, USDA 126, USDA 127, USDA 129 and USDA 135 were not reported. The sequences were initially aligned by using the software MEGA, version 5 [67]. Subsequently the alignment was manually inspected for errors and necessary corrections were made by using GeneDoc version 2.6.001 [68]. The outgroups *Mesorhizobium loti* LMG6125^T^ and *M. ciceri* UPM-Ca7^T^ were chosen because of the reported recombination events between the 16S rRNA genes of *B. elkanii* and *Mesorhizobium* [22]*.* Of the 1313 active sites of the alignment there were 40 gaps among the *Bradyrhizobium* taxa. The number of different base pairs among all the 35 aligned sequences (including the two *Mesorhizobium* species) was determined by using MEGA, version 5 [67] to generate a tree using the UPGMA algorithm. Bootstrap analysis [69] with 2000 permutations of the data set was used to determine support for each of the branches. Type strains are indicated by name in the figure. Strains in the figure with a genome sequencing project registered in GOLD [70] are as follows: *B. daqingense* (2596849087), USDA 110 (640700549), USDA 76 (2513649183), USDA 6 (2513666035), *B. pachyrhizi* (2655289729), and *B. yuanmingense* (2617374406)

Sequence divergence among the 16S rRNA genes of the 33 type strains within the genus *Bradyrhizobium* was limited and ranged from no differences in many cases to a similarity of 98% between *B. elkanii* USDA 76^T^ and *B. neotropicale* (Fig. 2) after accounting for 40 bp in gaps along the alignment length. Such high similarity values would question the reliability of defining species limits within the genus based on divergence of the 16S rRNA genes [19]. Bootstrap values for each of the nodes of the branches were low and none of the confidence values reached or exceeded 95%. Therefore, the placement of each of the taxa relative to the others in the tree is inconclusive.

Genetic recombination resulting in a reticulate evolutionary history of the 16S rRNA gene is perhaps a likely explanation for the low bootstrap values. Therefore, an analysis for recombination was done with the aligned 33 *Bradyrhizobium* 16S rRNA genes using the pairwise homoplasy index test [20]. By using this test, statistically significant evidence for recombination among the 33 16S rRNA genes was detected (*P* = 0.003). The detection of genetic recombination within the *rrn* loci of rhizobia is not unprecedented since reticulate evolutionary histories of the 16S rRNA genes and the Internally Transcribed Spacer between the 16S and 23S rRNA genes has been described before [21, 22]. The 16S rRNA sequence of *B. pachyrhizi* was identical with those of the *B. elkanii* serogroup strains USDA 31, USDA 94 and USDA 130, which differed from *B. elkanii* USDA 76^T^ by one bp (99.999% similar). The most divergent 16S rRNA gene within *B. elkanii* was that of the serogroup strain USDA 46 (99.996% similar), while the most divergence among the soybean serogroup strains was that between USDA 46 and USDA 110, which were 98.4% similar. Since the divergence of the 16S rRNA genes of the genus *Bradyrhizobium* is narrow, with evidence for the presence of a history of genetic recombination, it may be necessary to more precisely establish their phylogeny by comparing their entire genomes rather than individual genes. Such an approach may provide more fundamental insight into the evolutionary history of this class of symbiotic bacteria as well as impacting potential changes in their current proposed taxonomy. Minimum Information about the Genome Sequence of USDA 76^T^ is provided in Table 1 and Additional file 1: Table S1.

**Table 1:** Classification and general features of *Bradyrhizobium elkanii* USDA 76^T^ in accordance with the MIGS recommendations [71] published by the Genome Standards Consortium [72]

MIGS ID	Property	Term	Evidence code
	Classification	Domain Bacteria	TAS [73]
		Phylum *Proteobacteria*	TAS [74, 75]
		Class *Alphaproteobacteria*	TAS [74, 76]
		Order *Rhizobiales*	TAS [77]
		Family *Bradyrhizobiaceae*	TAS [78]
		Genus *Bradyrhizobium*	TAS [8, 78]
		Species *elkanii*	IDA
	Gram stain	Negative	IDA
	Cell shape	Rod	IDA
	Motility	Motile	IDA
	Sporulation	Non-sporulating	NAS
	Temperature range	Mesophile	NAS
	Optimum temperature	28°C	NAS
	pH range; Optimum	Unknown	NAS
	Carbon source	Arabinose, gluconate	TAS [17]
MIGS-6	Habitat	Soil, root nodule of *Glycine max* (L. Merr)	NAS
MIGS-6.3	Salinity	0 to <2% (w/v) NaCl	TAS [78]
MIGS-22	Oxygen requirement	Aerobic	NAS
MIGS-15	Biotic relationship	Free living, symbiotic	TAS
MIGS-14	Pathogenicity	Non-pathogenic	TAS [79]
MIGS-4	Geographic location	Alexandria, Virginia, USA	NAS
MIGS-5	Sample collection date	1915	NAS
MIGS-4.1	Latitude	38.8047	NAS
MIGS-4.2	Longitude	−77.0472	NAS
MIGS-4.3	Depth	5 cm	NAS
MIGS-4.4	Altitude	13 m	NAS

Evidence codes–*IDA* Inferred from Direct Assay, *TAS* Traceable Author Statement (i.e., a direct report exists in the literature), *NAS* Non-traceable Author Statement (i.e., not directly observed for the living, isolated sample, but based on a generally accepted property for the species, or anecdotal evidence). Evidence codes are from the Gene Ontology project [80, 81]

The original isolation location and date indicated is that of the parent culture USDA 8

#### Symbiotaxonomy

An investigation of the symbiotic properties of soybean began with the work of Brooks [23] in the late 19th century, when he observed that soybean grown in the fields of his experiment station in Massachusetts only nodulated when supplied with dust he had brought with him from Japan. This led to the theory that soybean-nodulating bacteria in the soils of the USA were imported from the Far East. Cotrell et al. [24] and Hopkins [25] reported the supporting evidence that soybean in Kansas nodulated with soil taken from the Massachusetts Experiment station, or in Illinois from soil collected from fields with a history of soybean cultivation. However, several decades later it became evident that rhizobia that nodulated native American legumes within the genera *Apios*, *Amphicarpa*, *Crotalaria*, *Desmodium*, *Lespedeza*, *Baptisia*, *Cassia*, *Genista* and *Wisteria* also nodulated soybean [26–28]. With the exception of USDA 6 and USDA 38, which are from Japan, all the remaining soybean serotype strains were recovered from nodules of soybeans grown in the USA, including USDA 8 (the original parent of USDA 76^T^). Consequently, it is unclear whether these rhizobia obtained from nodules of USA-grown soybean originate from the Far East or are in fact native to the soils of America. Therefore, the possibility exists that USDA 76^T^ may be able to nodulate and form a symbiosis with a wide variety of legumes, but this has not been thoroughly investigated. Unfortunately, the communication that included the proposal of USDA 76^T^ as the type strain for *B. elkanii* did not include results of plant tests to describe its symbiotic range, but instead relied on distinction by phenotype and genotype [11]. An indication of the possible American origin of USDA 76^T^ is its reported effectiveness in symbiosis with the native *Apios americana* Medik. and use as an inoculum for this potential leguminous crop [29]. Further evidence for this theory is the ability of USDA 76^T^ to nodulate and fix nitrogen with the native American *Amphicarpaea bracteata* (L.) Fernald [30]. USDA 76^T^ effectively nodulates the promiscuous *Vigna unguiculata* (L.) Walp. (cowpea), but is unable to nodulate the tropical American legume *Phaseolus lunatus* L. (Lima bean), which forms nodules with various other strains of bradyrhizobia [31]. To our knowledge, the only other reported information is that USDA 74 (parent of USDA 76^T^) forms an effective symbiosis with *Macroptilium atropurpureum* (DC.) Urb. (Siratro) and *Vigna unguiculata* (L.) Walp [32].

In soybean, the *Rj*(s) or *rj*(s) genetic loci have been identified as controlling the ability of compatible rhizobia to nodulate with a particular cultivar (reviewed by Hayashi et al. [33]). USDA 76^T^ is reported to form nodules (albeit in reduced numbers) on the cultivar Clark (*rj1*) and to nodulate and fix N_2_ with the isogenic lines BARC-2 and BARC-3, harboring the *Rj4* and *rj4* alleles, respectively, when tested in Leonard jars with sterile vermiculite or sand [30]. The symbiotic characteristics of *B. elkanii* USDA 76^T^ on a range of selected hosts are summarized in Additional file 2: Table S2.

## Genome sequencing information

### Genome project history

This organism was selected for sequencing at the U.S. Department of Energy funded Joint Genome Institute as part of the *Genomic Encyclopedia of* *Bacteria* *and Archaea-Root Nodule* *Bacteria* project project [14, 15]. The root nodule bacteria in this project were selected on the basis of environmental and agricultural relevance to issues in global carbon cycling, alternative energy production, and biogeochemical importance. In particular, strain USDA 76^T^ was chosen since it is a microsymbiont of the economically important legume soybean, but can also form symbioses with several legumes native to the USA. The USDA 76^T^ genome project is deposited in the Genomes Online Database [34] and a high-quality permanent draft genome sequence is deposited in IMG [35]. Sequencing, finishing and annotation were performed by the JGI [36] and a summary of the project information is shown in Table 2.

**Table 2:** Genome sequencing project information of *Bradyrhizobium elkanii* strain USDA 76^T^

MIGS ID	Property	Term
MIGS-31	Finishing quality	High-quality permanent draft
MIGS-28	Libraries used	2× Illumina libraries; Std short PE & CLIP long PE
MIGS-29	Sequencing platforms	Illumina HiSeq2000, PacBio
MIGS-31.2	Fold coverage	3,560×
MIGS-30	Assemblers	Velvet version 1.1.05; Allpaths-LG version r38445; phrap, version 4.24
MIGS-32	Gene calling methods	Prodigal 1.4; GenePRIMP
	Locus Tag	Brael [82]
	GenBank ID	ARAG00000000
	GenBank Date of Release	Apr 22, 2013
	GOLD ID	Gp0009610
	NCBI BIOPROJECT	162247
MIGS-13	Source Material Identifier	USDA 76, USDA 8, USDA 74, ATCC 49852, DSM 11554, IFO (now NBRC) 14791, LMG 6134
	Project relevance	Symbiotic N_2_ fixation, agriculture

### Growth conditions and genomic DNA preparation

After recovery from permanent storage, the *B. elkanii* USDA 76^T^ was streaked onto MAG solid medium and grown at 28 °C for 6 days to obtain well grown, well separated colonies, then a single colony was selected and used to inoculate 5 ml MAG broth. The culture was grown on a gyratory shaker (200 rpm) at 28 °C for 6 days. Subsequently 1 ml was used to inoculate 50 ml MAG broth and grown on a gyratory shaker (200 rpm) at 28 °C until an OD_600nm_ of 0.6 was reached. DNA was isolated from the cells according to van Berkum [17]. Final concentration of the DNA was set to 0.5 mg ml^−1^. Culture identity was confirmed by partial sequence analysis of several housekeeping genes and the 16S rRNA gene using the prepared DNA as template for PCR.

### Genome sequencing and assembly

The draft genome of *B. elkanii* USDA 76^T^ was generated at the DOE Joint genome Institute (JGI) using the Illumina technology [37]. An Illumina short-insert paired-end library was constructed with an average insert size of 200 bp that when sequenced generated 312,796,730 reads. An Illumina long-insert paired-end library with an average insert size of 6505.78 +/− 3679.88 bp also was constructed that when sequenced generated 19,315,434 reads. The total amount of sequence data obtained with the Illumina was 34,177 Mbp. Library construction and sequence analysis were done at the JGI according to the protocols outlined on their website [38]. The first of two initial drafts, assembled with Allpaths version r38445 [39], contained 81 contigs in 17 scaffolds and subsequently a consensus was computationally shredded into 10 Kbp overlapping fake reads (shreds). The second draft assembled with Velvet, version 1.1.05 [40], resulted in consensus sequences that were computationally shredded into 1.5 Kbp overlapping fake reads (shreds). The data were assembled again with Velvet using the shreds from the first Velvet assembly to guide the next assembly. The consensus from this second Velvet assembly was shredded into 1.5 Kbp overlapping fake reads. The fake reads from the Allpaths and both Velvet assemblies together with a subset of the Illumina CLIP paired-end reads were assembled using parallel Phrap, version 4.24 (High Performance Software, LLC). Potential errors in the assemblies were corrected by manual editing with Consed [41–43]. Gap closure was accomplished using repeat resolution software (Wei Gu, unpublished) and sequence analysis of bridging PCR fragments with PacBio technology (Cliff Han, unpublished). Gaps were closed and the quality of the final sequence was improved with 35 PCR PacBio consensus sequences. The total size of the genome is 9.5 Mbp and the final assembly is based on 34,177 Mbp of Illumina draft data, which provides an average 3560x coverage of the genome.

### Genome annotation

Genes were identified using Prodigal [44] that was followed by a round of manual curation using GenePRIMP [45] as part of the DOE-JGI genome annotation pipeline [46, 47]. The predicted CDSs were translated and used to search the National Center for Biotechnology Information (NCBI) non-redundant, UniProt, TIGRFam, Pfam, KEGG, COG, and InterPro databases. The tRNAScanSE tool [48] was used to find tRNA genes. Ribosomal RNA genes were found by searches against models of the ribosomal RNA genes built from SILVA [49]. Other non–coding RNAs such as the RNA components of the protein secretion complex and the RNase P were identified by searching the genome for the corresponding Rfam profiles using INFERNAL [50]. Additional gene prediction analysis and manual functional annotation were done within the Integrated Microbial Genomes-Expert Review system [51] developed by the Joint Genome Institute, Walnut Creek, CA, USA.

## Genome properties

The genome of *B. elkanii* USDA 76^T^ is 9,484,767 nucleotides long with a GC content of 63.70% (Table 3) and has been assembled into two scaffolds. Of the 9151 genes identified, 9060 are protein encoding and 91 are RNA only encoding genes. Of the 9151 total genes identified in USDA 76^T^, the majority (73.28%) were assigned a putative function and the remaining genes were annotated as hypothetical. The distribution of genes into COGs functional categories is presented in Table 4.

**Table 3:** Genome statistics for *Bradyrhizobium elkanii* USDA 76^T^

Attribute	Value	% of Total
Genome size (bp)	9,484,767	100.00
DNA coding (bp)	8,070,200	85.09
DNA G + C (bp)	6,041,732	63.70
DNA scaffolds	2	100.00
Total genes	9151	100.00
Protein coding genes	9060	99.01
RNA genes	91	0.99
Pseudo genes	408	4.46
Genes in internal clusters	789	8.62
Genes with function prediction	6706	73.28
Genes assigned to COGs	5665	61.91
Genes with Pfam domains	7004	76.54
Genes with signal peptides	864	9.44
Genes with transmembrane helices	2055	22.46
CRISPR repeats	2

**Table 4:** Number of protein coding genes of *Bradyrhizobium elkanii* USDA 76^T^ associated with the general COG functional categories

Code	Value	Percent	COG Category
J	235	3.63	Translation, ribosomal structure and biogenesis
A	0	0.00	RNA processing and modification
K	514	7.93	Transcription
L	175	2.70	Replication, recombination and repair
B	2	0.03	Chromatin structure and dynamics
D	40	0.62	Cell cycle control, cell division, chromosome partitioning
V	165	2.55	Defense mechanisms
T	253	3.90	Signal transduction mechanisms
M	313	4.83	Cell wall/membrane/envelope biogenesis
N	80	1.23	Cell motility
U	135	2.08	Intracellular trafficking, secretion, and vesicular transport
O	267	4.12	Posttranslational modification, protein turnover, chaperones
C	439	6.77	Energy production and conversion
G	392	6.05	Carbohydrate transport and metabolism
E	685	10.57	Amino acid transport and metabolism
F	94	1.45	Nucleotide transport and metabolism
H	317	4.89	Coenzyme transport and metabolism
I	423	6.53	Lipid transport and metabolism
P	381	5.88	Inorganic ion transport and metabolism
Q	295	4.55	Secondary metabolite biosynthesis, transport and catabolism
R	663	10.23	General function prediction only
S	399	6.16	Function unknown
-	3486	38.09	Not in COGS

## Insights from the genome sequence

**Fig. 3:**
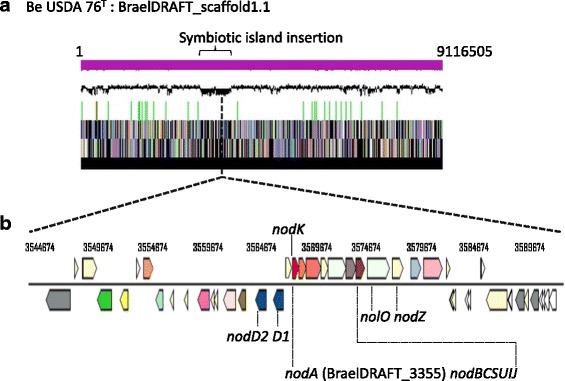
Graphical map of the largest scaffold (9,116,505 bp) of USDA 76^T^ (**a**) showing the location of common nodulation genes within the symbiotic island of this strain (**b**). From bottom to the top of the scaffold map: Genes on forward strand (color by COG categories as denoted by the IMG platform), genes on reverse strand (color by COG categories), RNA genes (tRNAs green, sRNAs red, other RNAs black), GC content, GC skew

**Fig. 4:**
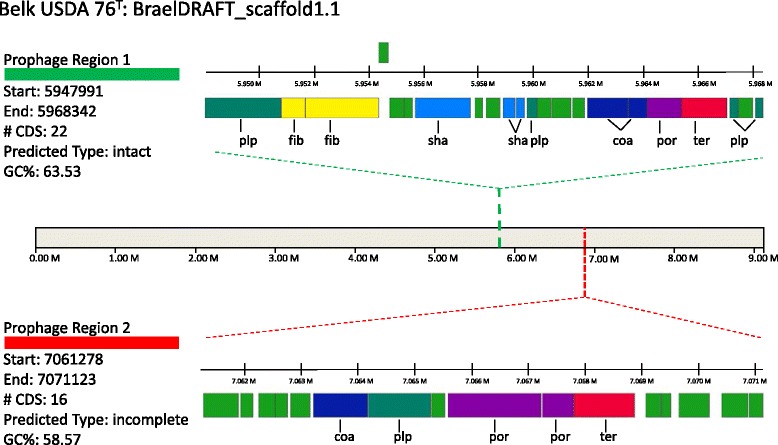
Resident prophage present in *Bradyrhizobium elkanii* USDA 76^T^ imaged using PHASTER [54]. Prophage maps are not drawn to scale. Reference locus tag for Prophage Region 1 is BraelDRAFT_5594 terminase; ter); reference locus tag for Prophage Region 2 is BraelDRAFT_6751 (terminase; ter). Coat protein (coa), fiber protein (fib), phage-like protein (plp), portal protein (por), tail shaft protein (sha), and terminase (ter). All other genes encode hypothetical proteins

Scaffold 1.1 of *B. elkanii* USDA 76^T^ contains a low GC content for the region ~3,000,000–3,800,000 and the presence of symbiotic *nod*, *nif* and *fix* genes in this region indicates a symbiotic island integration (Fig. 3). Using the Phylogenetic Profiler tool within IMG, 356 genes were found to be unique to USDA 76^T^ in a comparison with four other strains (587 [52], CCBAU43297, CCBAU05737 [53] and USDA 94) ascribed to the *B. elkanii* IMG clique. Of those that were unique, the majority (223 genes, representing 62.6%) were annotated as encoding hypothetical proteins. Out of the remainder, a significant number were phage related. Using the PHASTER algorithm [54], 22 of these genes were found to be co-located genes of an intact resident prophage (Fig. 4). Using this algorithm another incomplete phage gene set on the same scaffold was also identified.

### Extended insights

Using the Phylogenetic Profiler tool, 7556 genes were found to be conserved in five *B. elkanii* strains (587, CCBAU43297, CCBAU05737, USDA 76^T^, USDA 94), including genes encoding a general secretion pathway and type II, III, IV and VI secretion system proteins. The Type III secretion system (T3SS) [55] can either promote or impair the establishment of symbiosis, depending on the legume host [56], and has been characterized as a host determinant for *rj1, Rfg1, Rj2* and *Rj4* soybean cultivars [33, 57, 58]. The dominant soybean genes *Rj2* and *Rj4* restrict nodulation with specific strains of *Bradyrhizobium* [33]. Most investigations of soybean host genes controlling the symbiosis have focused on the *Rj4* soybean line that was originally identified by its inability to nodulate with USDA 61 (*B. elkanii*, serogroup 31) [59]. The predicted *Rj4* thaumatin-like protein is thought to be involved in conferring resistance to *Bradyrhizobium* strains producing specific T3SS effector proteins [60]. However, USDA 76^T^ was reported to nodulate and form an effective nitrogen-fixing symbiosis with the isogenic lines BARC-2 (*Rj4*) and BARC-3 (*rj4*) [30, 61], suggesting that this strain does not produce the interacting T3SS effector protein(s). Conversely, the recessive soybean gene *rj1rj1* [62], encoding a putative truncated Nod factor receptor protein [63], restricts nodulation by many *Bradyrhizobium* and *Ensifer* strains, although specific strains of *B. elkanii*, including USDA 76^T^, can form a limited number of nodules when tested with plants in Leonard jars containing sterilized vermiculite or sand [30, 59, 61].

**Fig. 5:**
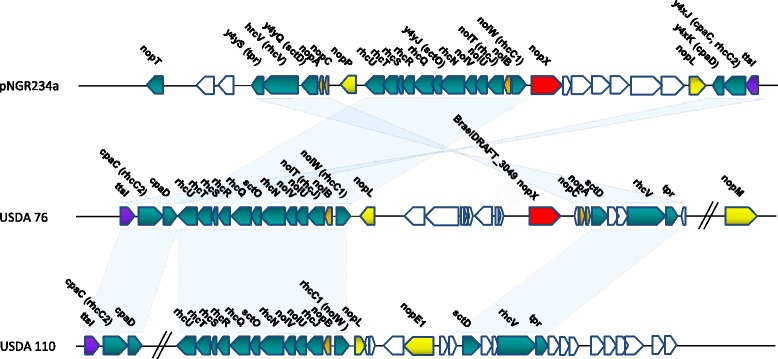
Comparison of the gene neighbourhood regions containing loci that encode type III secretion system components in the genomes of *Ensifer fredii* NGR234 and the *Bradyrhizobium* strains USDA 76^T^ and USDA 110. The colour scheme is as follows: *green*, structural component; *orange*, pilus component; *purple*, regulatory component; *red*, translocon component; *uncoloured*, other genes; and *yellow*, effector component

**Fig. 6:**
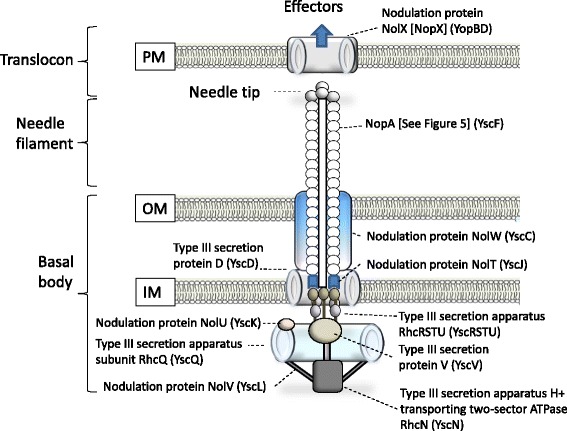
Schematic representation of the components constituting the T3SS present in *Bradyrhizobium elkanii* USDA 76^T^. The IMG product name is provided with the *Yersinia* Ysc-Yop T3SS ortholog shown in *brackets*. The relative secretion components were identified based on information provided by Galán et al. [55]

USDA 76^T^ genes encoding components required for a functional T3SS were identified within the integrated symbiotic island (Figs. 5 and 6). Although the *nopA* and *nopC* genes were not annotated in the USDA 76^T^ genome, by using TBLASTN these genes were identified in the intergenic region between BraelDRAFT_3047 (*sctD*) and BraelDRAFT_3048 (hypothetical) that share 100% sequence similarity with *nopA* and *nopC* of the characterized *Bradyrhizobium elkanii* strain USDA 61 [57]. Although T3SS components can also be found in *Bradyrhizobium* strain USDA 110, this strain lacks the *nopX* gene encoding the translocon required to introduce effector molecules into host cells [56, 64]. This is in contrast to the presence of *nopX* in USDA 76^T^, which could extend its host range to otherwise incompatible hosts.

## Conclusions

*B. elkanii* USDA 76^T^ originated from strain USDA 8, which was obtained in 1915 from an effective nodule of soybean grown on the USDA Arlington farm in Virginia. Its ability to nodulate the native North American legumes *Apios americana* Medik. and *Amphicarpaea bracteata* (L.) Fernald indicates a possible North American origin for this strain. USDA 76^T^ was selected for genome sequencing [14] because of its significance as a microsymbiont of soybean. The genome size of USDA 76^T^ was established as 9.5 Mbp, which falls within the range of 7.7 to 10.5 Mbp observed for other bradyrhizobial genomes. The genome of this N_2_-fixing microsymbiont contains *nod*, *nif* and *fix* genes located on an integrated symbiotic island, and genes encoding both an intact and an incomplete phage. According to ANI values, strain USDA 76^T^ formed an ANI clique with four other *B. elkanii* soybean strains: USDA 94, 587, CCBAU 43297 and CCBAU 05737. Of particular interest was the discovery that these strains contain a T3SS that contains the NopCA pilus genes and the NopX translocon protein, which are essential for introducing effector molecules into host cells [55]. The T3SS has been shown to be an important host range determinant that enables the nodulation of some soybean cultivars and is detrimental to symbiosis with other cultivars [56]. Here we postulate that the presence of a functional T3SS is important in determining the host range of USDA 76^T^ and enables it to form some nodules on the soybean cultivar Clark (*rj1*) when grown in Leonard jars with sterilized vermiculite or sand [65, 66]. Further analyses of *Bradyrhizobium* genomes, including that of USDA 76^T^, will increase our understanding of determinants that lead to the establishment and functioning of different *Bradyrhizobium* symbioses.

## Additional files

Additional file 1: Associated MIGS record. Table S1. Associated MIGS record for *Bradyrhizobium elkanii* USDA 76^T^. (DOCX 19 kb)
Additional file 2: Symbiotic properties of USDA 76^T^. Table S2. Nodulation and N_2_-fixation properties of *Bradyhizobium elkanii* USDA 76^T^ on selected legume hosts. (DOCX 16 kb)

## Abbreviations

½ LA: ½ Lupin Agar
ANI: Average Nucleotide Identity
GEBA-RNB: Genomic Encyclopedia for Bacteria and Archaea-Root Nodule Bacteria
IMG: Integrated Microbial Genomes
MAG: Modified Arabinose Gluconate
T3SS: Type 3 Secretion System
