# Supplementary material for: High-quality permanent draft genome sequence of the Bradyrhizobium elkanii type strain USDA 76T, isolated from Glycine max (L.) Merr
Source: Stand Genomic Sci. 2017 Mar 4;12:26. doi: 10.1186/s40793-017-0238-2 (PMC5336687; doi:10.1186/s40793-017-0238-2)
Supplement: Additional file 1: — Associated MIGS record. Table S1. Associated MIGS record for Bradyrhizobium elkanii USDA 76T. (DOCX 19 kb) [file 40793_2017_238_MOESM1_ESM.docx]

# Additional file 1: Associated MIGS record

**Table S1.** Associated MIGS record for *Bradyrhizobium elkanii* USDA 76^T^

| **MIGS-ID** | field name | description |
| --- | --- | --- |
| **MIGS-1** | Submit to INSDC/Trace archives |  |
| **1.1** | PID |  |
| **1.2** | Trace Archive |  |
| **MIGS-2** | MIGS CHECK LIST TYPE |  |
| **MIGS-3** | Project Name | GEBA Root Nodulating Bacteria |
| **MIGS-4** | Geographic Location | Virginia, USA |
| **4.1** | Latitude | 38.87997 |
| **4.2** | Longitude | -77.10677 |
| **4.3** | Depth | 5cm |
| **4.4** | Altitude | 84 m |
| **MIGS-5** | Time of Sample collection |  |
| **MIGS-6** | Habitat (EnvO) | Soil, root nodule, host |
| **6.1** | temperature | 28 |
| **6.2** | pH |  |
| **6.3** | salinity |  |
| **6.4** | chlorophyll |  |
| **6.5** | conductivity |  |
|  |  |  |
| **6.6** | light intensity |  |
| **6.7** | dissolved organic carbon (DOC) |  |
| **6.8** | current |  |
| **6.9** | atmospheric data |  |
| **6.10** | density |  |
| **6.11** | alkalinity |  |
| **6.12** | dissolved oxygen |  |
| **6.13** | particulate organic carbon (POC) |  |
| **6.14** | phosphate |  |
| **6.15** | nitrate |  |
| **6.16** | sulfates |  |
| **6.17** | sulfides |  |
| **6.18** | primary production |  |
| **MIGS-7** | Subspecific genetic lineage |  |
| **MIGS-9** | Number of replicons |  |
| **MIGS-10** | Extrachromosomal elements |  |
| **MIGS-11** | Estimated Size |  |
| **MIGS-12** | Reference for biomaterial or Genome report |  |
| **MIGS-13** | Source material identifiers |  |
| **MIGS-14** | Known Pathogenicity | Non-pathogen |
|  |  |  |
| **MIGS-15** | Biotic Relationship | Symbiotic |
| **MIGS-16** | Specific Host | *Glycine max* |
| **MIGS-17** | Host specificity or range (taxid) |  |
| **MIGS-18** | Health status of Host |  |
| **MIGS-19** | Trophic Level |  |
| **MIGS-22** | Relationship to Oxygen | Aerobe |
| **MIGS-23** | Isolation and Growth conditions | TY media, 28°C, aerobe |
| **MIGS-27** | Nucleic acid preparation | CTAB |
| **MIGS-28** | Library construction | IlluminaStd short PE & CLIP long PE |
| **28.1** | Library size | 34,177 Mbp |
| **28.2** | Number of reads | Std short PE reads totaling 312,796,730 and CLIP long PE reads totaling 19,315,434 |
| **28.3** | vector |  |
| **MIGS-29** | Sequencing method | Illumina HiSeq2000,PacBio |
| **MIGS-30** | Assembly |  |
| **30.1** | Assembly method | Velvet version 1.1.05; Allpaths-LG version r38445; phrap, version 4.24 |
| **30.2** | estimated error rate |  |
| **30.3** | method of calculation |  |
| **MIGS-31** | Finishing strategy |  |
| **31.1** | Status | High-quality permanent Draft |
| **31.2** | coverage | 3,560x |
| **31.3** | contigs | 25 |
| **MIGS-32** | Relevant SOPs |  |
| **MIGS-33** | Relevant e-resources |  |
