# Supplementary material for: High-quality permanent draft genome sequence of the Bradyrhizobium elkanii type strain USDA 76T, isolated from Glycine max (L.) Merr
Source: Stand Genomic Sci. 2017 Mar 4;12:26. doi: 10.1186/s40793-017-0238-2 (PMC5336687; doi:10.1186/s40793-017-0238-2)
Supplement: Additional file 2: — Symbiotic properties of USDA 76T. Table S2. Nodulation and N2-fixation properties of Bradyhizobium elkanii USDA 76T on selected legume hosts. (DOCX 16 kb) [file 40793_2017_238_MOESM2_ESM.docx]

# Additional file 2: Symbiotic properties of USDA 76^T^

Table S2. Nodulation and N_2_-fixation properties of *Bradyhizobium elkanii* USDA 76^T^ on selected legume hosts.

| **Species Name** | **Cultivar** | ***Rj* (*rj*) allele** | **Nodulation phenotype** | **Reference** |
| --- | --- | --- | --- | --- |
| *Amphicarpaea bracteata* (L.) Fernald | NL^a^ | ND | Nod+ Fix+ | [45] |
| *Amphicarpaea bracteata* (L.) Fernald | JWC^a^ | ND | Nod+ Fix+ | [45] |
| *Apios americana* Medik. |  |  | Nod+ Fix+ | [44] |
| *Glycine max* (L.) Merr. | Clark | *Rj1* | Nod+ Fix+ | [45] |
| *Glycine max* (L.) Merr. | Clark | *rj1* | Sparse nodulation | [45] |
| *Glycine max* (L.) Merr. | BARC-2 | *Rj4* | Nod+ Fix+ | [45] |
| *Glycine max* (L.) Merr. | BARC-3 | *rj4* | Nod+ Fix+ | [45] |
| *Macroptilium atropurpureum* (DC.) |  | ND | Nod+ Fix+ | [47]^b^ |
| *Phaseolus lunatus* L. |  | ND | Nod- | [46] |
| *Vigna unguiculata* (L.) Walp. |  | ND | Nod+ Fix+ | [46] |

^a^ Cultivar NL is symbiotically specific; cultivar JWC is a symbiotic generalist

^b^ Nod+Fix+ with the parent strain USDA 74.
